# How does the modular organization of entorhinal grid cells develop?

**DOI:** 10.3389/fnhum.2014.00337

**Published:** 2014-06-03

**Authors:** Praveen K. Pilly, Stephen Grossberg

**Affiliations:** ^1^Information and Systems Sciences Laboratory, HRL Laboratories, LLC, Center for Neural and Emergent SystemsMalibu, CA, USA; ^2^Department of Mathematics, Center for Adaptive Systems, Graduate Program in Cognitive and Neural Systems, Center for Computational Neuroscience and Neural Technology, Boston UniversityBoston, MA, USA

**Keywords:** grid cells, module, navigation, self-organizing map, recurrent inhibition, temporal integration, oscillatory interference, continuous attractor

## Abstract

The entorhinal-hippocampal system plays a crucial role in spatial cognition and navigation. Since the discovery of grid cells in layer II of medial entorhinal cortex (MEC), several types of models have been proposed to explain their development and operation; namely, continuous attractor network models, oscillatory interference models, and self-organizing map (SOM) models. Recent experiments revealing the *in vivo* intracellular signatures of grid cells (Domnisoru et al., 2013; Schmidt-Heiber and Hausser, 2013), the primarily inhibitory recurrent connectivity of grid cells (Couey et al., 2013; Pastoll et al., 2013), and the topographic organization of grid cells within anatomically overlapping modules of multiple spatial scales along the dorsoventral axis of MEC (Stensola et al., 2012) provide strong constraints and challenges to existing grid cell models. This article provides a computational explanation for how MEC cells can emerge through learning with grid cell properties in modular structures. Within this SOM model, grid cells with different rates of temporal integration learn modular properties with different spatial scales. Model grid cells learn in response to inputs from multiple scales of directionally-selective stripe cells (Krupic et al., 2012; Mhatre et al., 2012) that perform path integration of the linear velocities that are experienced during navigation. Slower rates of grid cell temporal integration support learned associations with stripe cells of larger scales. The explanatory and predictive capabilities of the three types of grid cell models are comparatively analyzed in light of recent data to illustrate how the SOM model overcomes problems that other types of models have not yet handled.

## Introduction

During navigation in the external world, the brains of many animals are able to update representations of their current position, or place. While the underlying neural computations involved still remain to be fully elucidated, place cells in the hippocampus (O'Keefe and Dostrovsky, 1971) and grid cells in parahippocampal areas, including the medial entorhinal cortex (MEC) (Hafting et al., 2005; Sargolini et al., 2006), presubiculum (PrS), and parasubiculum (PaS) (Boccara et al., 2010), are understood to play critical roles. These space-encoding cells respond both to displacements from a reference position as well as to environmental sensory stimuli.

Stensola et al. (2012) recently performed a comprehensive study of the anatomical organization of two-dimensional grid scales in layers II and III of MEC, both within and across animals. Their experiments showed that grid cells along the dorsoventral axis of MEC have a modular organization; namely, that grid cells along the dorsoventral axis “cluster into a small number of layer-spanning anatomically overlapping modules with distinct scale, orientation, asymmetry, and theta-frequency modulation” (p. 72). In other words, these grid cell modules are distributed along the dorsoventral extent with the different modules overlapping significantly. This article describes a neural model that proposes how such a modular organization may arise through learning as an animal navigates in its environment during postnatal development.

It had earlier been shown that average grid cell properties vary in a systematic way along the dorsoventral axis. In particular, the rate of temporal integration along the dorsoventral axis of MEC layer II decreases (Garden et al., 2008) while the grid cell spatial scale increases from the dorsal to the ventral end (Brun et al., 2008). There is also a systematic decrement in the frequency of subthreshold membrane potential oscillations (MPOs) along the dorsoventral extent of MEC layer II (Giocomo et al., 2007; Yoshida et al., 2011). Grossberg and Pilly (2012) and Pilly and Grossberg (2013b) described a neural model in which a suitably designed self-organizing map (SOM) develops the observed properties during navigation of realistic rat trajectories; see below.

The GRIDSmap model (Mhatre et al., 2012) and its refinements and extensions in the GridPlaceMap model (Pilly and Grossberg, 2012, 2013a) and the Spectral Spacing model (Grossberg and Pilly, 2012; Pilly and Grossberg, 2013b) predicted how inputs to MEC are provided by ensembles of *stripe cells* that perform path integration of the linear velocities that are experienced during navigation. Stripe cells are predicted to be computational homologs of head direction cells (Ranck, 1984; Blair and Sharp, 1995; Taube, 1995; Redish et al., 1996; Stackman and Taube, 1997) that perform path integration of the angular velocities that are experienced during navigation, notably during head turns.

Both stripe cells and head direction cells are predicted to occur in ring attractor circuits. Different stripe cells in a given ring attractor respond at different spatial phases, and multiple stripe cell ring attractors are proposed to exist, each corresponding to stripe cells with preference for a given direction and spatial scale. In particular, a ring attractor that responds to linear velocity along an allocentric direction results in stripe cells that have spatial firing fields resembling regularly spaced parallel stripes that are perpendicular to the corresponding direction, hence the term “stripe cells.”

The coding of spatial position based on path integration is implicit in the ensemble responses of stripe cells. Why then does the brain need grid cells and place cells? Previous modeling work has suggested that grid and place cells occur because they arise naturally in a hierarchy of self-organizing maps (SOMs) through the MEC and hippocampal cortex (HC), responding to stripe cell inputs (Grossberg and Pilly, 2012; Pilly and Grossberg, 2012). The place cells that are learned have large enough scales to represent behaviorally relevant spaces (Gorchetchnikov and Grossberg, 2007), and output explicit spatial (position) information to frontal and motor circuits involved in planning and executing navigational movements through space. Both grid cells and place cells in the SOM models learn to adapt the strengths of their inputs to gradually become selective for a subset of input patterns that are the most frequent and energetic (Pilly and Grossberg, 2012). Grossberg and Pilly (2012) showed in addition that the gradient, from fast to slow, in the rate of temporal integration along the dorsoventral axis of MEC layer II (Garden et al., 2008) can drive the development of grid cells whose spatial scales increase from the dorsal to the ventral end (Brun et al., 2008) in response to inputs from stripe cells of multiple scales. Specifically, map cells with faster response rates preferentially learn from stripe cell input subsets with smaller scales, whereas those with slower response rates choose larger scales. The temporal integration rate gradient also accounts for, as epiphenomena, the observed variations in the frequency of subthreshold membrane potential oscillations (MPOs) along the dorsoventral extent of MEC layer II (Giocomo et al., 2007; Yoshida et al., 2011); also see Dodson et al. (2011). Grossberg and Pilly (2012) thus showed the presence of these MPOs need not imply a causal role for them in grid cell firing, as some authors have assumed (e.g., Burgess et al., 2007; Giocomo et al., 2007; Hasselmo et al., 2007).

As noted above, Stensola et al. (2012) provided a comprehensive analysis of the anatomical organization of grid cells. They reported that grid cell scales are grouped into finitely many *modules* such that the cells in each module share some defining characteristics. In particular, grid cells that share similar scales also share similar grid orientations, and are modulated at similar theta band frequencies in their interspike interval histograms. Moreover, grid cells belonging to the same module, rather than different modules, show similarity in their rescaling responses to environmental compression along a dimension. Finally, grid cells grouped by similar attributes are not locally clustered, but are distributed with significant anatomical overlap among the modules along the dorsoventral axis (see Figure 4A).

If indeed grid cells develop from path integration inputs that are mediated by stripe cells, then the data of Stensola et al. (2012) implies that the problem of selecting from multiple scales of stripe cells during early development is a real one, if only because a simple topographic mapping from stripe cells to grid cells with no interference across scales is not consistent with these data. These modular constraints significantly challenge existing grid cell models. For example, how are recurrent connections in the MEC pruned so that nearby grid cells belonging to different modules do not interact functionally? This article shows that a refinement of the SOM model in Grossberg and Pilly (2012) can account for anatomically overlapping grid modules. In particular, the SOM model assumes that the map cells at a given dorsoventral location exhibit a range of temporal integration rates, and that these rates at more ventral locations are sampled from a relatively broader tuning function that prefers slower values. In contrast, the response rates of map cells in Grossberg and Pilly (2012) are the same at each dorsoventral location. The Discussion section provides a detailed comparative analysis of various grid cell models in light of recent data, including the data about modular organization (e.g., Stensola et al., 2012; Couey et al., 2013; Domnisoru et al., 2013; Pastoll et al., 2013; Schmidt-Heiber and Hausser, 2013; Yoon et al., 2013). The new simulation results presented in this article are primarily aimed at providing elaborations for the Figure 6 presented in Grossberg and Pilly (2014).

## Methods

The first simulation study tested how the temporal integration rate gradient of MEC cells (Garden et al., 2008) influences grid cell development when there is no “scale selection” problem, i.e., when only one scale of stripe cells generates inputs during the initial phase of spatial experience. The development of a SOM comprising 25 map cells across 20 learning trials was simulated in response to stripe cells of a particular scale (either *s* = 20, 35, or 50 cm) and for various cell response rates (μ = 0.1, 0.2, 0.3, 0.4, 0.5, 0.6, 0.7, 0.8, 0.9, or 1). Note that μ is a dimensionless parameter that scales the rate of temporal integration. In each trial the model animal ran in a 100 cm wide circular box starting at the midpoint and along a novel realistic rat trajectory for about 20 min. The instantaneous linear velocity and head direction extracted from the trajectory were used to algorithmically compute the activities of the input stripe cells (see Equations 1–4). Thirty SOM simulations were performed to systematically assess the importance of the temporal response rate of map cells emerging into grid cells of a given spatial scale, in terms of mean gridness score, mean grid spacing, mean inter-trial stability, and the proportion of map cells that are classifiable as grid cells (i.e., with gridness score > 0.3).

The second study investigated how multiple grid scales are learned by map cells within a single SOM (i.e., a local cell population), when they have distributed cell response rates *μ*. The development of three SOMs was simulated: one comprising 50 map cells, all with response rate *μ* = 1, in response to inputs from stripe cells of two spacings (*s*_1_ = 20, *s*_2_ = 35 cm); one comprising 50 cells, half with *μ* = 1 and the remaining with *μ* = 0.6, in response to inputs from stripe cells of two spacings (*s*_1_ = 20, *s*_2_ = 35 cm); and another comprising 90 cells, one-third with *μ* = 1, one-third with *μ* = 0.6, and the remaining with *μ* = 0.3, in response to inputs from stripe cells of three spacings (*s*_1_ = 20, *s*_2_ = 35, *s*_3_ = 50 cm). The SOM equations and parameters that were used are provided in the Appendix. In particular, Equation 6 describes the competitive instar learning rule (Grossberg, 1976; Carpenter and Grossberg, 1987; Grossberg and Seitz, 2003), which accounts for activity-dependent pruning of input weights to each map cell (e.g., Tsanov and Manahan-Vaughan, 2007), while automatically conserving the total adaptive weight to each map cell (e.g., Royer and Pare, 2003), that underlies the gradual development of tuning of different map cells to the different sets of input features that are experienced through time.

## Results

**Figure 1:**
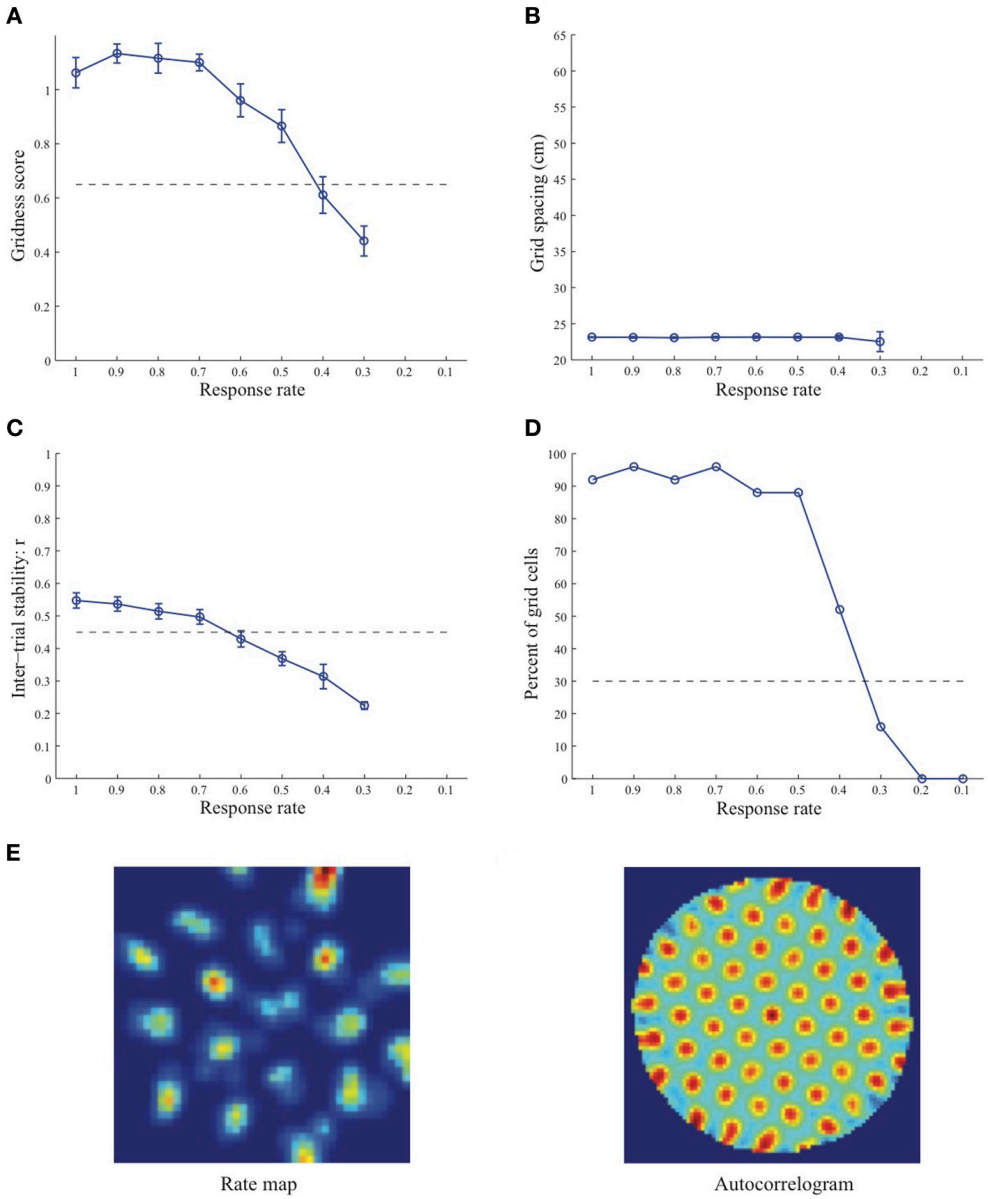
**Properties of learned grid cells in the SOM model as a function of response rate μ, responding to single-scale stripe cell inputs with a spacing *s* = 20 cm**. Panels **(A–D)** show gridness score, grid spacing, inter-trial stability, and the proportion of learned grid cells (with gridness score > 0.3), respectively. Peak activity *A_s_* of stripe cells was 1. Each error bar in **(A–C)** corresponds to standard error of mean (s.e.m.). The dashed lines parallel to the x-axis in **(A)**, **(C)**, and **(D)** signify corresponding experimentally measured values for adult dorsal grid cells (Langston et al., 2010; Wills et al., 2010). Panel **(E)** shows the spatial rate map and autocorrelogram of the learned grid cell with the highest gridness score in the last trial (#20) in the map corresponding to the optimal response rate *μ* = 0.9. Color coding from blue (min.) to red (max.) is used for the rate map, and from blue (−1) to red (1) for the autocorrelogram.

**Figure 2:**
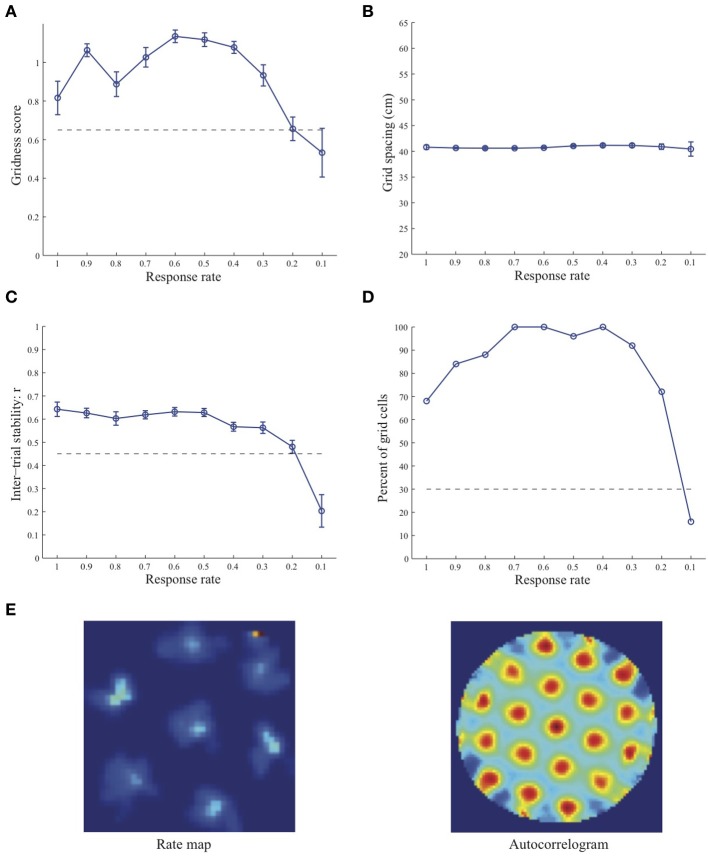
**Properties of learned grid cells in the SOM model as a function of response rate μ, responding to single-scale stripe cell inputs with a spacing *s* = 35 cm**. Panels **(A–D)** show gridness score, grid spacing, inter-trial stability, and the proportion of learned grid cells (with gridness score > 0.3), respectively. Peak activity *A_s_* of stripe cells was 1. Each error bar in **(A–C)** corresponds to standard error of mean (s.e.m.). The dashed lines parallel to the x-axis in **(A)**, **(C)**, and **(D)** signify corresponding experimentally measured values for adult dorsal grid cells (Langston et al., 2010; Wills et al., 2010). Panel **(E)** shows the spatial rate map and autocorrelogram of the learned grid cell with the highest gridness score in the last trial (#20) in the map corresponding to the optimal response rate *μ* = 0.6. Color coding from blue (min.) to red (max.) is used for the rate map, and from blue (−1) to red (1) for the autocorrelogram.

**Figure 3:**
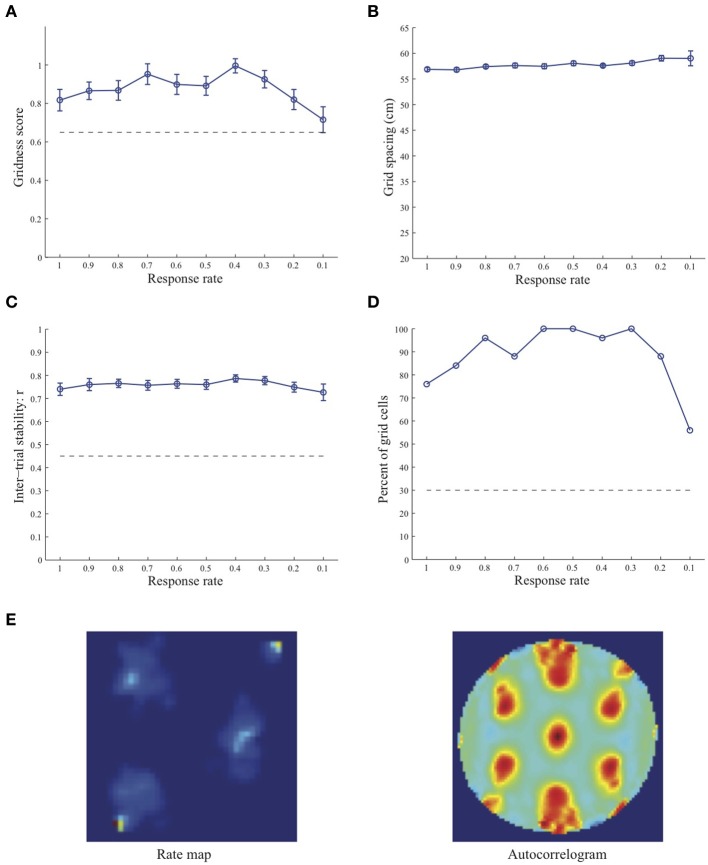
**Properties of learned grid cells in the SOM model as a function of response rate μ, responding to single-scale stripe cell inputs with a spacing *s* = 50 cm**. Panels **(A–D)** show gridness score, grid spacing, inter-trial stability, and the proportion of learned grid cells (with gridness score > 0.3), respectively. Peak activity *A_s_* of stripe cells was 1. Each error bar in **(A–C)** corresponds to standard error of mean (s.e.m.). The dashed lines parallel to the x-axis in **(A)**, **(C)**, and **(D)** signify corresponding experimentally measured values for adult dorsal grid cells (Langston et al., 2010; Wills et al., 2010). Panel **(E)** shows the spatial rate map and autocorrelogram of the learned grid cell with the highest gridness score in the last trial (#20) in the map corresponding to the optimal response rate *μ* = 0.4. Color coding from blue (min.) to red (max.) is used for the rate map, and from blue (−1) to red (1) for the autocorrelogram.

Simulation results presented in Figures 1–3 reveal that the optimal *temporal* response rate μ for grid cell learning depends on the *spatial* scale, even when the input stripe cells have the same scale. In particular, the smaller the grid spacing, the larger is the optimal response rate. For example, for the input stripe spacing *s* = 20 cm the mean gridness score of the learned map cells peaks at *μ* = 0.9 (Figure 1A), whereas for the input stripe spacing *s* = 50 cm the corresponding peak occurs at *μ* = 0.4 (Figure 3A). Similarly, the mean inter-trial stability and the proportion of learned grid cells exhibit similar trends in their tuning to response rate μ. Moreover, the tuning widths also increase with the spatial scale. These results provide further support to our previously described hypothesis that the rate of temporal integration of entorhinal map cells determines the subset of input stripe scales to which they can get tuned, and thereby the development of their regular hexagonal grid fields (Grossberg and Pilly, 2012). Also, whereas the mean gridness score at the optimal response rate is relatively smaller for bigger spatial scales, the mean spatial stability is larger, consistent with experimental observations (Giocomo et al., 2011).

When a map cell fires intensely at a given spatial position, it undergoes activity-dependent adaptation. The cell response rate controls not only the dynamics of adaptation but also the temporal duration during which the cell recovers from adaptation to be able to fire intensely again. In particular, the recovery period is inversely proportional to the response rate (see Figure 3 in Grossberg and Pilly, 2012). Given that learning is turned on at all times, it would be ideal for grid cell learning if the map cell were to become excitable again just when the model animal arrives at one of the nearest vertices of the corresponding grid as it navigates in the environment. The emerging strong input weights from the appropriate stripe cell combination (i.e., three stripe cells of the corresponding scale whose preferred directions differ from each other by 60° that are consistently coactive during map cell activation) could be lost, or recoded, if the cell becomes excitable too early or too late. The mean linear and rotational velocities, even though they change in a non-stationary way, seem to determine the average interval between visits of the navigating animal to any vertex of an arbitrary grid. Note that the mean running speed of juvenile rats is not significantly different from that of adult rats (Langston et al., 2010). These factors combine to determine scale-dependent optimal response rates for better, more stable, and greater number of grid cells. Along the same lines, the tuning widths of learned grid cells as a function of response rate μ can be understood as consequences of how slowly the cell excitability recovers from habituation, and the average temporal extent of map cell activation during passage through a grid vertex.

**Figure 4:**
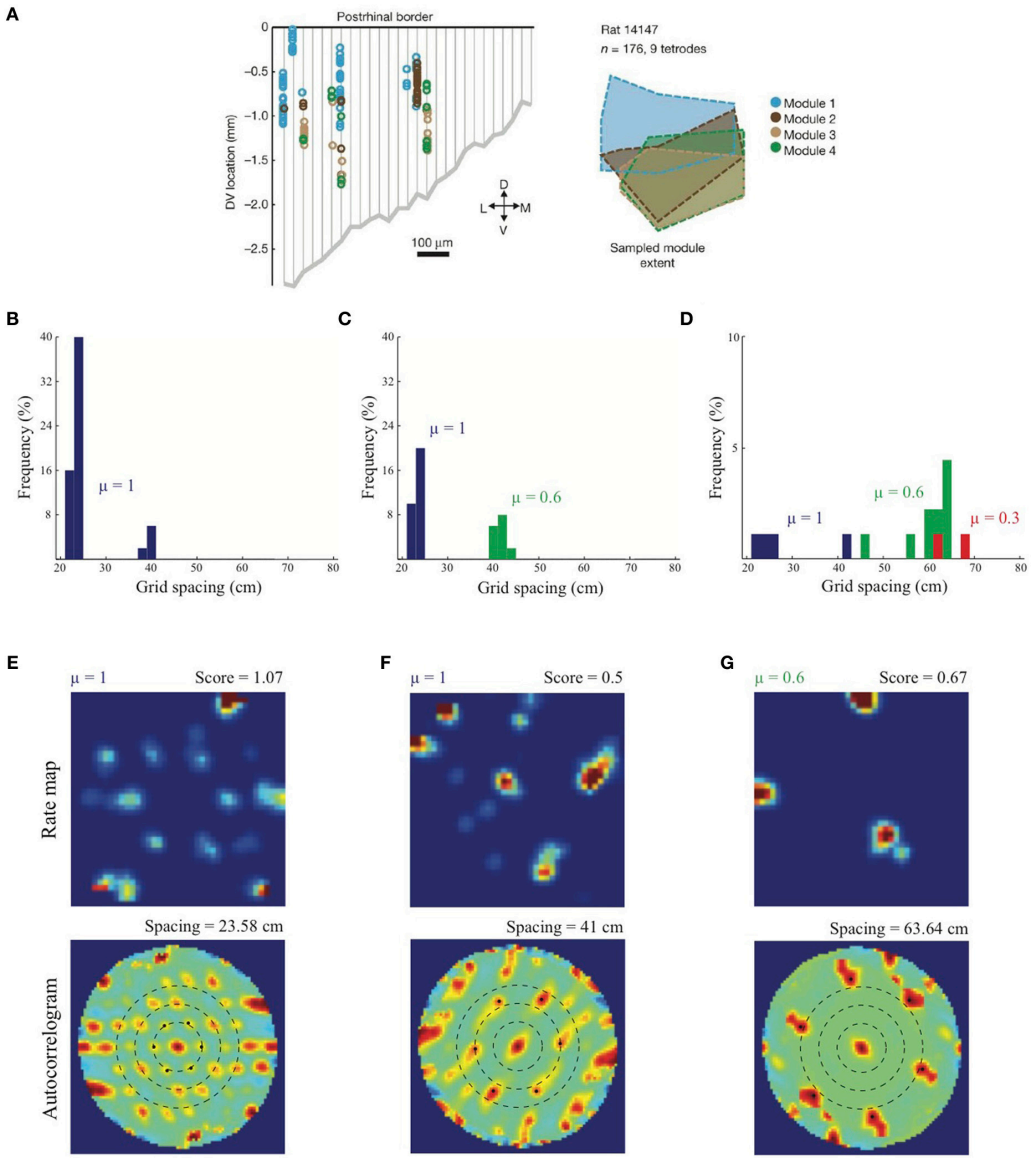
**Anatomically overlapping grid cell modules. (A)** Anatomical distribution of sampled grid cells belonging to different modules in one animal (Stensola et al., 2012). Simulation results of the SOM model: **(B)** Distribution of learned grid spacings in a SOM comprising 50 map cells, all with response rate *μ* =1, that are receiving adaptive inputs from stripe cells of two spacings (*s*_1_ = 20 cm, *s*_2_ = 35 cm). Only cells with gridness score > 0.3 are considered. **(C)** Distribution of learned grid spacings in a SOM comprising 50 cells, half with *μ* =1 and the remaining with *μ* =0.6, that are receiving adaptive inputs from stripe cells of two spacings (*s*_1_ = 20 cm, *s*_2_ = 35 cm). **(D)** Distribution of learned grid spacings in a SOM comprising 90 cells, one-third with *μ* =1, one-third with *μ* = 0.6, and the remaining with *μ* = 0.3, that are receiving adaptive inputs from stripe cells of three spacings (*s*_1_ = 20 cm, *s*_2_ = 35 cm, *s*_3_ = 50 cm). Panels **(E–G)** show spatial rate maps and autocorrelograms of illustrative grid cells with different learned spacings from the simulation summarized in **(D)**. Note response rate (μ) and gridness score at the top of each rate map, and grid spacing at the top of each autocorrelogram. Peak activities *A_s_* of stripe cells were 1, 0.8, 0.6 for spacings of 20, 35, 50 cm, respectively (see Equation 4). Color coding from blue (min.) to red (max.) is used in each rate map, and from blue (−1) to red (1) in each autocorrelogram. [Data in **(A)** is reprinted with permission from Stensola et al. (2012), and the other panels with permission from Grossberg and Pilly (2014)].

Simulation results presented in Figure 4B replicate the main finding of Grossberg and Pilly (2012) that the response rate μ shared by all map cells within an entorhinal SOM can bias the selection among input stripe cell scales. In particular, the faster response rate of *μ* = 1 causes most of the learned grid cells to choose the smaller of the two input scales (*s*_1_ = 20, *s*_2_ = 35 cm). Simulation results in Figures 4C,D demonstrate for the first time the learning of multiple grid scales (up to three) within the same local network of map cells that recurrently inhibit each other and vary in their response rates μ. Figure 4C shows grid cells with two scales emerging from competing map cells that have faster (*μ* = 1) and slower (*μ* = 0.6) response rates, respectively, in response to input stripe cells of two spacings (*s*_1_ = 20, *s*_2_ = 35 cm). And Figure 4D shows that in response to three input stripe scales (*s*_1_ = 20, *s*_2_ = 35, *s*_3_ = 50 cm) a subset of cells in the local network with the faster response rate (*μ* = 1) develop into grid cells with the smaller two of the three scales, while a subset of those with the medium response rate (*μ* = 0.6) develop into grid cells with the larger two of the three scales. This shows that cells with the same temporal rate can learn multiple spatial scales, which is consistent with Stensola et al. (2012)'s observation that the “temporal organization does not exhibit any strong linear or monotonic relationship to grid spacing” (p. 76). Figures 4E–G show spatial rate maps and autocorrelograms of illustrative grid cells with different learned spacings from the simulation summarized in Figure 4D. Note that the peak activities *A_s_* of stripe cells in Equation 4 decrease with spatial scale (see Simulation Settings) to balance the competitive advantage of associative learning during longer temporal intervals for weights from stripe cells of larger scales [cf., Figure 13C in (Grossberg and Pilly, 2012)]. The new model results about distributed response rates within local ensembles are consistent with experiments showing spreads in different intrinsic properties of MEC layer II stellate cells at given dorsoventral locations (e.g., Giocomo et al., 2007; Garden et al., 2008; Boehlen et al., 2010; Navratilova et al., 2012).

## Discussion

### Mechanisms of grid cell module formation

Sensory cortical circuits develop both *in utero* and during the postnatal critical period in response to statistical regularities in the stream of signals from the sensory organs. For example, the development of ocular dominance columns and selectivity to different features (such as location, spatial frequency, orientation, and direction) in the primary visual cortex of several species has been well documented (e.g., Hubel and Wiesel, 1962, 1977; Payne et al., 1981; Weliky et al., 1996). The current modeling results are consistent with a similar idea in the development of spatial representation, notably that genetic and morphogenetic laws are complemented by developmental and learning processes that are sensitive to correlations among afferent signals generated during early navigational experiences to shape space-encoding neural circuits in the brain. This view contrasts with that stated in Stensola et al. (2012, p. 72): “These [hippocampal and MEC] maps are different from sensory maps in that spatial firing fields are not derived by extraction of features from a particular sensory input, but probably originate from pattern-formation processes in the circuit itself. The mechanisms for topographical organization may thus be very dissimilar from those of the columnar sensory cortices.” Although SOM models also include “pattern-formation processes” whereby their recurrent competitive interactions respond to the statistics of input patterns through time, there is a real conceptual difference among the models that are under discussion. In particular, among the envisaged pattern-formation processes are models that are called continuous attractor network (CAN) models that have been used to propose an explanation of how grid cell responses change after focal hippocampal inactivation (Bonnevie et al., 2013). Grossberg and Pilly (2014) have summarized conceptual and data-explanatory problems with the CAN account of these data, and have provided an alternative explanation that is compatible with SOM dynamics. See below for a ***Comparative model analysis***.

The main contribution of this article is to provide an explanation for how nearby grid cells that are mutually interacting within the same local network can develop to exhibit different spatial scales. The SOM model predicts that the temporal integration rates of MEC layer II stellate cells exhibit significant local variation, which biases them to become selective for preferred sets of stripe cell inputs of different spacings. Indeed, the optimal *temporal* integration rate of model grid cells, with respect to hexagonal gridness quality, spatial stability, and proportion of learned grid cells, is inversely proportional to their *spatial* scale, even though running speed and head direction are widely distributed and change in a non-stationary manner through time. Future research needs to investigate the robustness of grid cell learning to noisy stripe cells and noisy linear and angular velocity estimates, and a possible role for adaptive inhibitory connections (Couey et al., 2013) in the development of functionally independent grid cell modules that overlap spatially (Stensola et al., 2012). In this regard, it should be noted that the contrast-enhancing properties of the SOM model's competitive interactions are, among other things, designed to suppress noise (Grossberg, 1980).

### Two outstanding questions

The current results raise at least two questions that need further research to answer:

1. A re there computational advantages, with respect to learning better hippocampal place codes, of the topographic organization of grid cell scales revealed by Stensola et al. (2012)? In particular, if multiple grid scales are available at a given location along the dorsoventral axis of MEC, then why do convergent perforant path projections to the hippocampus from several locations exist (Dolorfo and Amaral, 1998)? Or are modules just an unavoidable emergent property of a coarsely defined temporal integration rate gradient along the MEC dorsoventral axis?
2. What are the mechanisms by which stripe cells develop to support the path integration of linear velocity along different allocentric directions? Knowing this will place additional constraints on the self-organization of grid cells and place cells, and will enable a comprehensive study of top-down interactions to be made. The proposal that there is a ring attractor organization both of stripe cells, for linear velocity integration, and of head direction cells, for angular velocity integration, is parsimonious, but how do these ring attractors develop? Fortenberry et al. (2012) have modeled how the calibration of visual, vestibular, and motor inputs develops within the head direction ring attractor, but not how the ring attractor itself develops.

### Comparative model analysis

CAN models simulate the two-dimensional spatial periodicity of grid cell responses using a recurrent neural network. While several variations have been proposed (see Zilli, 2012), they all generate a stable attractor state of cell activations when the animal is stationary and a shift in the attractor's response pattern as a result of movements in external space. While some variants use short-range recurrent excitation and long-range recurrent inhibition to support a localized bump of persistent activity in the neural plane (2D: Guanella et al., 2007; Pastoll et al., 2013; 1D: Navratilova et al., 2012), others use a non-specific tonic excitatory drive combined with recurrent inhibition for the encoding of stationary spatial position (Burak and Fiete, 2009; Bonnevie et al., 2013; Couey et al., 2013). The former subclass of CAN models assume periodic boundaries (i.e., ring/toroidal topology) and employ either asymmetric inhibition by differential activation of various conjunctive (grid × head direction) cells during movement (Conklin and Eliasmith, 2005; Navratilova et al., 2012), or velocity-controlled asymmetry in recurrent connectivity (Guanella et al., 2007) to explain the spatially periodic activations of grid cells through time. The hexagonal grid structure, instead of a rectangular one, in their spatial responses emerges by appropriately twisting the toroidal arrangement of the network cells (Guanella et al., 2007).

In the latter subclass of CAN models each cell receives an excitatory input proportional to the component of body velocity along its preferred direction, and asymmetric recurrent inhibition that is offset in its preferred direction. The strength and extent of recurrent inhibitory connections determine the grid field size and spacing. Burak and Fiete (2009) investigated periodic and aperiodic instantiations of this two-dimensional network, which are related to whether or not the outbound recurrent connections near each edge wrap around to the opposite edge. While periodic CANs are more robust path integrators, aperiodic CANs can be designed to exhibit comparable performance by tapering the strength of feedforward inputs at locations closer to the boundaries, and increasing the size of the network (i.e., number of cells). Fundamental predictions of these models are the presence of ensembles of distinct grid cells that code the same spatial position (corresponding to multiple bumps), and the presence of preferred direction-specific offsets in recurrent inhibitory connectivity.

Despite lack of direct evidence for these critical assumptions, this subclass of CAN models has been promoted as the “best” among the existing ones in several recent experimental articles (Bonnevie et al., 2013; Couey et al., 2013; Domnisoru et al., 2013; Schmidt-Heiber and Hausser, 2013) based on their reports of experimental properties that are also shared with the SOM model. For example, stellate cells in layer II of MEC interact with each other not via recurrent excitatory connections but primarily through recurrent inhibition (Beed et al., 2013; Couey et al., 2013; Pastoll et al., 2013). Pastoll et al. (2013) proposed a variation of the Guanella et al. (2007) model in which recurrent connections among grid cells are exclusively inhibitory. In SOM models of grid cell and place cell firing (Pilly and Grossberg, 2012, 2013a), map cells at both the entorhinal and hippocampal levels also interact in a purely recurrent inhibitory network (see Equation 5). In addition, Beed et al. (2013) found that average spatial spread and the number of inhibitory interneurons (mainly parvalbumin positive) for a given stellate cell decreases from the dorsal to the ventral end. It is likely this dorsoventral gradient in recurrent inhibition further facilitates the learning of grid cell modules (Stensola et al., 2012), which needs to be studied in future work.

Further, Bonnevie et al. (2013) interpreted the disruption of the characteristic firing patterns of grid cells by hippocampal inactivation as evidence for a primary role in grid cell generation of spatially uniform and tonically active hippocampal excitatory inputs to them, in conflict with experimental evidence that hippocampal-to-entorhinal feedback signals are neither spatially uniform nor tonically active (see Grossberg and Pilly, 2014 for further discussion). Unlike the CAN models, neighboring place and grid cells in SOM models (Pilly and Grossberg, 2012, 2013a) can have spatial firing fields that are uncorrelated to their anatomical arrangement (Redish et al., 1996; Hafting et al., 2005). Moreover, the data of Stensola et al. (2012) that anatomically nearby grid cells can belong to different scale-specific modules, whose simulation and explanation by the SOM model are presented in this article, is especially challenging for CAN models because it raises the unavoidable issue of how such cells may be developmentally segregated into different attractor networks.

In this regard, while a recent study provided evidence for attractor dynamics underlying grid cell firing using simultaneous recordings to reveal stable relative spatial phases for grid cell pairs with similar spacings under environmental novelty and resizing (Yoon et al., 2013), it also clarified that more work is in order to differentiate a potential 2D CAN mechanism from one that receives inputs with 1D attractor dynamics (p. 1084). For the SOM model to directly simulate the coherent relative responses of grid cells (Yoon et al., 2013), it will need to incorporate external inputs that are sensitive to environmental features.

Domnisoru et al. (2013) and Schmidt-Heiber and Hausser (2013) used *in vivo* whole-cell recordings during virtual reality navigation to conclude that the firing of grid cells is better explained by membrane potential ramps caused by integration of synaptic inputs on a slower, sub-theta time scale, and not by constructive interference among intrinsic theta-band membrane potential oscillations (MPOs). Whereas these data may argue against oscillatory interference models (Burgess et al., 2007; Hasselmo et al., 2007), they are consistent with the SOM model. Further, as mentioned in the Introduction, Grossberg and Pilly (2012) showed that the frequency gradient of subthreshold MPOs of stellate cells along the dorsoventral extent of MEC layer II (Giocomo et al., 2007; Yoshida et al., 2011), which is regularly cited in support of oscillatory interference models (e.g., Burgess et al., 2007; Giocomo et al., 2007; Hasselmo et al., 2007), can also be accounted in the SOM model by the gradient in average cell response rates (Garden et al., 2008).

### Conflict of interest statement

The authors declare that the research was conducted in the absence of any commercial or financial relationships that could be construed as a potential conflict of interest.

## Appendix

This section describes the SOM model (Grossberg and Pilly, 2012) equations and parameters that were used in the simulations of grid cell module properties.

### Stripe cells

Stripe cells are algorithmically computed, for simplicity, as follows: If at time *t* the animat heads along allocentric direction φ(*t*) with velocity *v(t)*, then the velocity *v_d_(t)* along direction *d* is:

(1) $$v_{d}(t)=\cos(d-\varphi (t))v(t)$$

The displacement *D_d_(t)* traversed along direction *d* with respect to the initial position is calculated by path integration of the corresponding velocity:

(2) $$D_{d}(t)=\int_{0}^{t}v_{d}(\tau )d\tau$$

This directional displacement variable is converted into activations of various stripe cells. Let *S_dps_(t)* be the activity of a stripe cell whose spatial fields are oriented perpendicular to direction *d* with spatial phase *p* and spatial period *s*. *S_dps_(t)* will be maximal at periodic positions *ns* + *p* along direction *d*, for all integer values of *n*. In other words, *S_dps_(t)* will be maximal whenever (*D_d_* modulo *s*) = p. Defining the spatial phase difference ω_*dps*_ between *D_d_* and *p* with respect to spatial scale *s* by:

(3) $$\omega_{dps}(t)=\left(D_{d}(t)-p\right)\text{ modulo }s,$$

the stripe cell activity *S_dps_(t)* is modeled by a Gaussian tuning function:

(4) $$S_{dps}(t)=A_{s}\cdot \exp\left(-\frac{{\left(\min\left(\omega_{dps}\left(t\right),s-\omega_{dps}\left(t\right)\right)\right)}^{2}}{2\sigma_{s}^{2}}\right),$$

where *A_s_* is the maximal activity and *σ*_*s*_ is the standard deviation of each of its individual stripe fields along preferred direction *d*. All directional displacement variables *D_d_(t)* were initialized to 0 at the start of each learning trial.

### Map cells

The membrane potential *V^m^_j_* of the MEC layer II map cell *j* in local population *m* obeys membrane equation, or shunting, dynamics within a recurrent on-center off-surround network (Grossberg, 1976, 1980) as follows:

(5) $$\begin{array}{l} \frac{dV_{j}^{m}}{dt}=10\mu_{j}[-AV_{j}^{m}+\left(B-V_{j}^{m}\right)\\ \;\left(\sum_{dps}w_{dpsj}^{m}x_{dps}+\alpha {\left({\left[V_{j}^{m}\right]}^{+}\right)}^{2}z_{j}^{m}\right)\\ \;-\left(C+V_{j}^{m}\right)\sum_{k\neq j}\beta {\left({\left[V_{k}^{m}-\Gamma \right]}^{+}\right)}^{2}], \end{array}$$

where *μ*_*j*_ controls the rate of temporal integration, also called the response rate, of the cell; *A* is the decay parameter corresponding to the leak conductance; *B* and −*C* are the reversal potentials of the excitatory and inhibitory channels, respectively; *w^m^_dpsj_* is the synaptic weight of the projection from the stripe cell with activity *S_dps_* in Equation 4 to the map cell *j* in population *m*; $\alpha {\left({\left[V_{j}^{m}\right]}^{+}\right)}^{2}$ is the on-center self-excitatory feedback signal of the cell, which helps to resolve the competition among map cells within cell population *m*, where [*V*]^+^ = max (*V*,0) defines a threshold-linear function, and α is the gain coefficient; *z^m^_j_* is the habituative transmitter gate of map cell *j*; and *β* is the connection strength of the inhibitory feedback signal ${\left({\left[V_{k}^{m}-\Gamma \right]}^{+}\right)}^{2}$ from map cell *k* in the off-surround to map cell *j* within population *m*. The output signal of map cell *j* is also ${\left({\left[V_{j}^{m}-\Gamma \right]}^{+}\right)}^{2}$. The membrane potential of each map cell was initialized to 0 at the start of each trial.

### Adaptive weights

The adaptive weights *w^m^_dpsj_* of projections from stripe cells to map cells are governed by a variant of the competitive instar learning law (Grossberg, 1976; Carpenter and Grossberg, 1987; Grossberg and Seitz, 2003):

(6) $$\begin{array}{l} \frac{dw_{dpsj}^{m}}{dt}=\lambda {\left({\left[V_{j}^{m}-\Gamma \right]}^{+}\right)}^{2}\\ \;\left[\left(1-w_{dpsj}^{m}\right)x_{dps}-w_{dpsj}^{m}\sum_{\left(p,q,r\right)\;\neq \;\left(d,p,s\right)}x_{pqr}\right], \end{array}$$

where *λ* is the learning rate; the map cell output signal ${\left({\left[V_{j}^{m}-\Gamma \right]}^{+}\right)}^{2}$ gates learning on and off; and the learning rule defines a self-normalizing competition among afferent synaptic weights to the target cell, leading to a maximum learned total weight to the cell of 1. Each weight *w^m^_dpsj_* was initialized to a random value drawn from a uniform distribution between 0 and 0.1 at the start of the first trial.

### Habituative gating

The habituative transmitter *z^m^_j_* of map cell *j* in population *m* is defined by:

(7) $$\frac{dz_{j}^{m}}{dt}=10\eta \left[\left(1-z_{j}^{m}\right)-\gamma z_{j}^{m}{\left(\alpha {\left({\left[V_{j}^{m}\right]}^{+}\right)}^{2}\right)}^{2}\right],$$

where *η* controls the overall response, or habituation, rate of the transmitter and *γ* scales its depletion rate. In particular, term $\left(1-z_{j}^{m}\right)$ controls the gate recovery rate to the target level of 1, and term $-\gamma z_{j}^{m}{\left(\alpha {\left({\left[V_{j}^{m}\right]}^{+}\right)}^{2}\right)}^{2}$ controls the gate inactivation rate, which is proportional to the current gate strength *z^m^_j_* times the square of the signal $\left(\alpha {\left({\left[V_{j}^{m}\right]}^{+}\right)}^{2}\right)$ that *z^m^_j_* gates in Equation 5. The squaring operation causes the gated signal to first increase and then decrease through time in response to excitatory input (Gaudiano and Grossberg, 1991), thereby regulating the duration of intense cell activity, and thus cell perseveration. The habituative transmitter of each map cell was initialized to its maximum value of 1 at the start of each trial.

### Simulation settings

The parameter values used in the simulations were *A* = 3; *B* = 1; *C* = 0.5; *α* = 17.5; *β* = 1.5; *γ* = 0.2; *λ* = 0.025; η = 0.05; and Γ = 0.1. The differential equations governing model dynamics were numerically integrated using Euler's forward method with a fixed time step Δ*t* = 2 ms.

The development of three entorhinal SOMs were simulated: one comprising 50 map cells, all with response rate *μ* = 1, that received adaptive inputs from stripe cells of two spacings (*s*_1_ = 20, *s*_2_ = 35 cm); one comprising 50 cells, half with *μ* = 1 and the remaining with *μ* = 0.6, that received adaptive inputs from stripe cells of two spacings (*s*_1_ = 20, *s*_2_ = 35 cm); and the other comprising 90 cells, one-third with *μ* = 1, one-third with *μ* = 0.6, and the remaining one-third with *μ* = 0.3, that received adaptive inputs from stripe cells of three spacings (*s*_1_ = 20, *s*_2_ = 35, *s*_3_ = 50 cm). In each case, stripe cells also varied with nine direction preferences (−80° to 80° in steps of 20°), and four spatial phases (*p* = [0, *s*/4, *s*/2, 3*s*/4] for the stripe spacing *s*) per direction. Peak activities *A_s_* of stripe cells were set to 1, 0.8, and 0.6 for spacings of 20, 35, and 50 cm, respectively. The standard deviation σ_*s*_ of each stripe field Gaussian tuning was set to 8.84% of the stripe spacing. The development of the entorhinal map cells into their adult counterparts was accomplished by employing 20 trials, in each of which the animat ran along a novel realistic trajectory of ~20 min in a circular environment with a radius of 50 cm. These trajectories were obtained by rotating an original rat trajectory (data: Sargolini et al., 2006) about the midpoint of the environment, which is also the starting point, by random angles. The original trajectory was, also, interpolated to increase its temporal resolution to match the time step of numerical integration of model dynamics (Δ*t* = 2 ms).

### Post-processing

The 100 × 100 cm environment was divided into 2.5 × 2.5 cm bins. During each trial, the amount of time spent by the animat in the various bins was tracked. The output activity of each map cell in every spatial bin was accumulated as the trajectory visited that bin. The occupancy and activity maps were smoothed using a 5 × 5 Gaussian kernel with standard deviation equal to one. At the end of each trial, smoothed rate maps for each map cell were obtained by dividing the cumulative activity variable by cumulative occupancy variable in each bin. For each map cell, six local maxima with *r*>0.05 and closest to the central peak in the spatial autocorrelogram of its smoothed rate map were identified. Gridness score, which measures how hexagonal and periodic a grid pattern is, was then derived using the method described in Wills et al. (2010), and grid spacing was obtained as the median of the distances of the six local maxima from the central peak (Hafting et al., 2005).
